# Anti-DFS70 Antibodies Are Associated With Proliferative Lupus Nephritis and Renal Pathological Activity

**DOI:** 10.3389/fimmu.2022.810639

**Published:** 2022-02-03

**Authors:** Dandan Chen, Li Zhao, Yingxin Dai, Fang Du, Enling Li, Xiangyu Niu, Zhiqing Wang, Bing Zheng, Liangjing Lu

**Affiliations:** ^1^ Department of Rheumatology, Renji Hospital, School of Medicine, Shanghai Jiao Tong University, Shanghai, China; ^2^ Department of Laboratory Medicine, Renji Hospital, School of Medicine, Shanghai Jiao Tong University, Shanghai, China

**Keywords:** activity Index (AI), anti-DFS70 antibodies, anti-dsDNA antibodies, antinuclear antibodies (ANA), lupus nephritis (LN)

## Abstract

**Objective:**

The significance of anti-dense fine speckles 70 (DFS70) antibodies in systemic lupus erythematosus (SLE) is still unclear, especially in lupus nephritis (LN) patients. We investigated the prevalence, clinical and pathological relevance of anti-DFS70 antibodies in LN patients.

**Methods:**

Anti-DFS70 antibodies were measured using enzyme-linked immunosorbent assays in 377 biopsy-proven LN patients, 268 non-LN SLE patients, 232 chronic kidney disease (CKD) patients, and 78 healthy individuals (HI). Demographic, clinical, and pathological parameters were compared between LN patients with and without anti-DFS70 antibodies. Stepwise multivariable logistic regression was performed to identify covariates associated with anti-DFS70 antibodies.

**Results:**

The prevalence of anti-DFS70 antibodies in LN (19.6%) was comparable to non-LN SLE patients (19.8%, *P*=0.9630), but was significantly higher than CKD patients (13.4%, *P*=0.0468) and HI (9.0%, *P*=0.0252). Using multivariable logistic regression analysis, the titer of anti-double-stranded DNA (dsDNA) antibodies (adjusted odds ratio=1.002, 95% confidence interval 1.001-1.003, *P*=0.004) was associated with positive anti-DFS70 antibodies in LN patients. In addition, anti-DFS70 antibodies were more prevalent in proliferative LN (22.0%, 68/309) compared to membrane LN patients (10.2%, 6/59, *P*=0.0376). Furthermore, LN patients with positive anti-DFS70 antibodies had significantly higher activity index (AI) compared to patients who were negative (8.0 vs 6.0, *P*=0.0131). However, the chronicity index was similar between the groups (3.0 vs 3.0, *P*=0.8412).

**Conclusion:**

Anti-DFS70 antibodies were not associated with LN development in SLE patients but were associated with anti-dsDNA antibodies, proliferative LN, and renal AI. This suggests their potential to serve as a non-histological biomarker for LN subclass and activity status.

## Introduction

The presence of antinuclear antibodies (ANA) recognizing intracellular antigens is a hallmark of lupus nephritis (LN), the most frequent manifestation of systemic lupus erythematosus (SLE) resulting in increased morbidity and mortality (1, 2). Of interest, antibodies against dense fine speckles 70 (DFS70), a nuclear antigen that has been identified as a DNA binding transcription co-activator P75 (3), and lens epithelium-derived growth factor (4), are an immunological paradox. Previous studies (5–7) have reported a higher prevalence of anti-DFS70 in healthy individuals (HI) compared to SLE patients. Anti-DFS70 antibodies could be used to exclude the diagnosis of SLE in patients (5) because the frequency of monospecific anti-DFS70 antibodies in SLE patients was reported between 0.4% to 3.1% (6–8), as well as the absence of anti-DFS70-positivity in HI developing SLE after 5 years of follow-up (9).

Anti-DFS70 antibodies in SLE patients are usually accompanied by pathogenic anti-extractable nuclear antigen (ENA) and anti-double-stranded DNA (dsDNA) antibodies (5, 6, 8, 10). In the last decade, numerous studies have focused on the serological and clinical relevance of anti-DFS70 antibodies in SLE patients (5–7, 10). To date, no defined autoantibodies or clinical manifestations have been found to be associated with anti-DFS70 antibodies (7). In a recent paper, Aljadeff et al. (11) reported that anti-DFS70 antibodies purified from HI tempered renal progression in lupus-prone mice. They inferred that anti-DFS70 antibodies potentially could protect against renal injury in SLE patients (11). However, clinical studies (5, 6, 10) did not find an association of anti-DFS70 antibodies with LN in SLE patients. Hence, additional work is required to determine the clinical significance of anti-DFS70 antibodies in LN development in SLE patients.

In this study, we investigated the prevalence of anti-DFS70 antibodies in a large Chinese cohort of biopsy-proven LN patients and compared them with non-LN SLE patients (NLN-SLE), chronic kidney disease (CKD) patients, and HI. Considering the lack of relevant studies that have investigated the clinical and pathological features of LN patients with positive anti-DFS70 antibodies, we analyzed the demographic, serological, and pathological relevance of anti-DFS70 antibodies in LN patients.

## Patients And Methods

### Patients

A total of 377 LN patients confirmed *via* renal biopsy were designated as the disease group. The three control groups that included 268 NLN-SLE, 232 CKD, and 78 age and gender-matched HI were enrolled in Renji hospital (Shanghai, China) to participate in this study. All SLE patients in this study were diagnosed based on the 1982 American College of Rheumatology (ACR) revised classification criteria (12). There are 44 LN and 41 NLN-SLE were newly onset SLE patients, which were diagnosed within 6 months and hadn’t received standard treatment at enrollment. Biopsy-proven LN were consecutively enrolled from October 2017 to March 2021. Patients who had active infections, pregnancy, or cancer were excluded from the study. Age and gender-matched HI were recruited by the Renji Hospital Physical Examination Center. HI had no history of autoimmune diseases, current infections, or cancer. Non-LN SLE patients were selected based on the absence of hematuria, pyuria, proteinuria, urinary casts, and decreased renal function as determined by their medical history. The CKD group consisted of 119 nephrotic syndrome (NS), 57 IgA nephropathy (IgAN), 35 primary membranous nephropathy (PMN), 11 diabetic nephropathy (DN), 6 purpuric nephritis (PPN), and 4 vasculitis-associated nephritis (VAN) patients.

Because serum samples were obtained from the residual samples in Clinical Laboratory Department, the requirement for informed consent was waived and the study was approved by the Institutional Review Board of Renji Hospital [No. (2017)201]. Demographic, clinical, and laboratory data were obtained from the patient’s medical records. Renal systemic lupus erythematosus disease activity index (rSLEDAI) is based on the sum value of the 4 components in SLEDAI and includes hematuria, pyuria, proteinuria, and urinary casts (13). LN patients were classified as active LN if rSLEDAI scores were ≥ 4 (14).

### Renal Histopathology

Renal biopsies were performed based on ACR recommendations (15). Renal biopsies were classified and reviewed by two experienced renal pathologists who were blinded to the study design. Proliferative LN (PLN) included patients with class III, class IV, class III + V, and class IV + V, and membranous LN (MLN) referred to patients who were primarily class V (16). The 2018 revised International Society of Nephrology/Renal Pathology Society (ISN/RPS) classification system (17) advocates the modified National Institutes of Health activity index (AI) and chronicity index (CI) to evaluate active and chronic status of LN patients, instead of using the shorthand A, A/C, and C subdivision in 2003 revised ISN/RPS classification (18). A total of 121 LN Patients who were diagnosed after the publication of the 2018 revised ISN/RPS classification had AI and CI in our study. Serum from LN patients was obtained from the Clinical Laboratory Department at the time of renal biopsy and stored at −80°C until use.

### Enzyme-Linked Immunosorbent Assay (ELISA)

The ELISA assay and optical density (O.D.) cutoff value for anti-DFS70 antibodies were performed as previously described (19). Anti-dsDNA and anti-C1q antibodies were quantified by ELISA using the CaptiaTM dsDNA kit (Trinity Biotech plc, Wick low, Ireland) and anti-C1q (IgG) kit (EUROIMMUN, Lübeck, Germany). Anti-nucleosome antibody titers were calculated based on fold change to standard controls using the anti-nucleosomes (IgG) kit (EUROIMMUN) following the manufacturer’s instruction. Anti-phospholipid antibodies were detected using Anti-Cardiolipin ELISA Kit (EUROIMMUN) and Anti-beta-2-Glycoprotein1 ELISA Kit (EUROIMMUN).

### Line Immunoblot Assay (LIA)

Anti-ENA antibodies to Sm, nRNP/Sm, SSA/Ro60, Ro52, SSB/La, histone, proliferative cell nuclear antigen (PCNA), and ribosomal P protein (Rib-p) were measured by LIA using the Euroline antinuclear antibody (ANA) Profile 3 kit (EUROIMMUN) according to the manufacturer’s instructions.

### Additional Laboratory Tests

Complement 3 (C3) and complement 4 (C4) were measured by immunonephelometric assays (Siemens Healthcare Diagnostics Inc., Newark, USA) according to the manufacturer’s instructions. Serum creatinine and 24 hour-urine protein levels were measured using Cygnus Auto CRE 7170 kits (Shino-test Corporation, Tokyo, Japan) and total protein UC FS kits (DiaSys Diagnostic Systems, Holzheim, Germany).

### Statistical Analysis

Statistical Package for Social Sciences (SPSS) version 24.0 (IBM-SPSS, Inc., Armonk, USA) and GraphPad Prism 7 software for Windows (GraphPad Software Inc., La Jolla, USA) were used to perform statistical analysis. Patient characteristics were summarized using descriptive statistics including mean ± standard deviation (SD) or median [interquartile range (IQR)]. To compare the characteristics between LN patients with and without anti-DFS70 antibodies, Student’s t-test or Mann-Whitney test was used for continuous variables and Fisher’s exact test or chi-square test (when appropriate) for categorical variables. Pearson’s correlation coefficient (*r* value) was used to identify the correlated continuous variables with the O.D. value of anti-DFS70 antibodies detected by ELISA. Spearman’s correlation coefficient (*r* value) was used to identify the correlated variables with AI and CI. A stepwise multivariable logistic regression model was constructed to identify covariates associated with positive anti-DFS70 antibodies. Covariates with a *P*-value < 0.05 in univariate logistic regression analysis were incorporated into the multivariable model. *P* values less than 0.05 were considered statistically significant.

## Results

### Prevalence and O.D. Values of Anti-DFS70 Antibodies in LN Patients and the Three Control Groups

Individuals in the LN (female: 87.8%) and HI (female: 87.2%) groups were predominantly females, with a mean age of 37.2 ± 12.6 and 39.5 ± 13.1 years, respectively. No significant differences in age and gender between LN and HI were observed. NLN-SLE patients had similar frequency of female patients (91.8%, *P*=0.1037) to LN patients, but they (40.0 ± 13.9 years, *P*=0.0066) were older compared to LN patients. Given that NS and IgAN typically affected elderly men (20–23), patients in the CKD group, which were mainly consisted of NS (51.3%, 119/232) and IgAN (24.6%, 57/232) patients, were predominantly males (53.5%), with a mean age of 50.7 ± 17.4 years.

The prevalence of anti-DFS70 antibodies in LN patients (19.6%, 74/377) was similar to NLN-SLE patients (19.8%, 53/268, *P*=0.9630), but significantly higher compared to HI (9.0%, 7/78, *P*=0.0252) and CKD patients (13.4%, 31/232, *P*=0.0468). Compared to NLN-SLE patients, LN patients had significantly higher prednisone dose, more hydroxychloroquine, mycophenolate mofetil, and other medication use except for the azathioprine (**Supplemental Table 1**). In addition, the disease duration of LN [5.0 (1.0, 10.0), years] was longer than NLN-SLE [3.0 (0.5, 10.0), years, *P*=0.0243] patients. To reduce the effect of different treatment strategies in NLN-SLE and LN patients for a more accurate anti-DFS70 prevalence assessment, we analyzed the positive rate of anti-DFS70 antibodies in newly onset NLN-SLE (n=41) and LN (n=44) patients. The positive rate of anti-DFS70 antibodies in these newly onset NLN-SLE (17.1%, 7/41) and LN (22.7%, 10/44, *P*=0.5149) patients remained comparable. Among the patients with CKD, 33.3% (2/6) PPN, 18.2% (2/11) DN, 15.8% (9/57) IgAN, 11.8% (14/119) NS, 11.4% (4/35) PMN and 0% (0/4) VAN were positive for anti-DFS70 antibodies. Contrary to the prevalence of anti-DFS70 antibodies, the frequency of isolated anti-DFS70 antibodies in HI (9.0%, 7/78, *P*=0.0003) and CKD patients (12.9%, 30/232, *P*=0.0001) were significantly higher compared to LN patients (0.8%, 3/377). Frequency of isolated anti-DFS70 antibodies (1.1%, 3/268, *P*=0.6967) in NLN-SLE patients was similar to LN patients.

**Figure 1:**
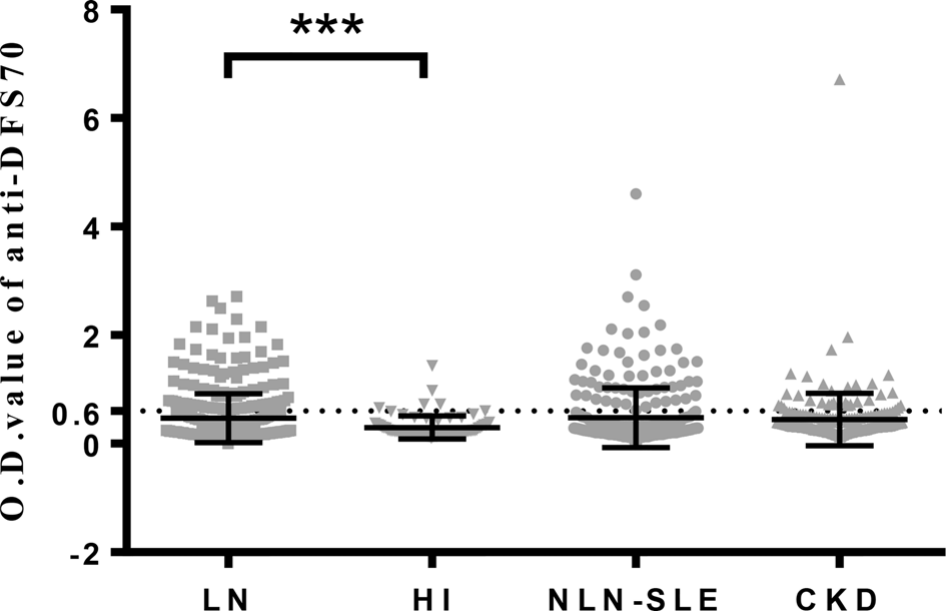
O.D. values of anti-DFS70 antibodies in LN patients and three control groups (HI, NLN-SLE and CKD) measured by ELISA. CKD, chronic kidney disease; DFS70, dense fine speckles 70; ELISA, enzyme-linked immunosorbent assay; HI, healthy individuals; LN, lupus nephritis; NLN-SLE, non-LN systemic lupus erythematosus; O.D. value, optical density value. The dotted line at 0.6 indicates the O.D. cutoff value for anti-DFS70 antibodies. ***P < 0.001.

O.D. values of anti-DFS70 antibodies detected by ELISA were also analyzed in LN patients and the three control groups (**Figure 1**). LN patients (0.47 ± 0.45) had significantly higher O.D. values compared to HI (0.30 ± 0.21, *P*=0.0009) and showed a higher trend compared to CKD patients (0.44 ± 0.48, *P*=0.0567). Nevertheless, O.D. values of anti-DFS70 antibodies in LN patients remained similar to NLN-SLE patients (0.48 ± 0.55, *P*=0.1310).

### Association of Anti-DFS70 Antibodies With Clinical Features in LN Patients

Comparisons of the clinical features of LN patients with and without anti-DFS70 antibodies are shown in **Table 1**. Using univariate logistic regression analysis, we found that LN patients with positive anti-DFS70 antibodies were younger compared to patients who were negative (34.5 vs 37.8 years, *P*=0.041). They also had significantly higher anti-dsDNA antibody titers (247.6 vs 172.2 IU/mL, *P*=0.001) and anti-nucleosome antibody titers (2.5 vs 1.6, *P*=0.016), higher positive rate of anti-histone antibodies (51.4% vs 29.7%, *P*=0.001) and lower levels of C4 (11.0 vs 13.9 mg/dL, *P*=0.035). The percentage of angiotensin-converting enzyme (ACE) inhibitors or aldosterone receptor blockers (ARB) use (59.5% vs 72.6%, *P*=0.027) was also lower in LN patients with positive anti-DFS70 antibodies compared to anti-DFS70-negative patients. These covariates with significant differences were included in stepwise multivariable analysis. Anti-dsDNA antibody titers [adjusted odds ratio (OR) =1.002, 95% confidence interval (CI) =1.001-1.003, *P*=0.004) remained associated with positive anti-DFS70 antibodies after multivariable adjustment (**Table 1**). Furthermore, the correlation coefficients between O.D. values for anti-DFS70 antibodies and the continuous covariates that had significant differences in the above univariate analysis including age, anti-dsDNA, anti-nucleosome, and C4 were also calculated (**Table 2**). In addition, comparing the positive rate of anti-DFS70 antibodies in the LN patients during active vs inactive disease, the positive rate of anti-DFS70 antibodies of active LN patients (20.6%, 64/310) was similar to inactive LN patients (14.8%, 9/61, *P*=0.2901).

**Table 1:** Comparisons of 377 LN patients with and without anti-DFS70 antibodies.

Parameter	anti-DFS70 Ab positive n=74	anti-DFS70 Ab negative n=303	Univariate analysis		Multivariable analysis	
			Unadjusted OR (95% CI)	*P* value	Adjusted OR (95% CI)	*P* value
Age, mean ± SD, years	34.5 ± 11.7	37.8 ± 12.7	0.978 (0.957-0.999)	**0.041**		
Gender, female, n (%)	70 (94.6)	261 (86.1)	2.816 (0.977-8.120)	0.055		
Duration of SLE, median (IQR), years	6.0 (2.0-10.0)	5.0 (1.0-10.0)	1.000 (0.961-1.040)	0.997		
SLEDAI-2K, median (IQR)	13.0 (8.0-16.0)	12.0 (6.0-16.0)	1.032 (0.993-1.074)	0.113		
rSLEDAI, median (IQR)	8.0 (4.0-12.0)	8.0 (4.0-12.0)	1.022 (0.967-1.080)	0.449		
Active LN^†^, n (%)	64 (86.5)	246 (81.2)	1.503 (0.704-3.212)	0.293		
Anti-dsDNA Ab, mean ± SD, IU/mL	247.6 ± 195.1	172.2 ± 167.5	1.002 (1.001-1.004)	**0.001**	1.002 (1.001-1.003)	**0.004**
Anti-nucleosome Ab, mean ± SD	2.5 ± 2.4	1.6 ± 2.5	1.132 (1.023-1.252)	**0.016**		
Anti-C1q Ab, n (%)	28 (37.8)	80 (26.4)	1.697 (0.994-2.896)	0.053		
Anti-C1q Ab, median (IQR), RU/mL	15.4 (3.5-38.6)	8.0 (3.2-23.5)	1.004 (0.999-1.009)	0.116		
Anti-histone Ab, n (%)	38 (51.4)	90 (29.7.)	2.463 (1.467-4.136)	**0.001**		
Anti-Sm Ab, n (%)	18 (24.3)	57 (18.8)	1.370 (0.749-2.508)	0.307		
Anti-nRNP/Sm Ab, n (%)	37 (50.0)	115 (38.0)	1.609 (0.964-2.683)	0.069		
Anti-SSA/Ro60 Ab, n (%)	42 (56.8)	166 (54.8)	1.059 (0.634-1.770)	0.825		
Anti-Ro52 Ab, n (%)	40 (54.1)	151 (49.8)	1.161 (0.697-1.933)	0.567		
Anti-SSB/La Ab, n (%)	7 (9.5)	28 (9.2)	1.015 (0.425-2.423)	0.973		
Anti-PCNA Ab, n (%)	3 (4.1)	7 (2.3)	1.769 (0.446-7.010)	0.417		
Anti-Rib-p Ab, n (%)	27 (36.5)	87 (28.7)	1.406 (0.824-2.401)	0.211		
Anti-cardiolipin Ab, n (%)	2 (2.7)	19 (6.3)	0.415 (0.095-1.824)	0.244		
Anti-beta-2-glycoprotein1 Ab, n (%)	2 (2.7)	27 (8.9)	0.284 (0.066-1.222)	0.091		
C3, mean ± SD, mg/dL	63.9 ± 26.6	70.9 ± 31.9	0.992 (0.983-1.001)	0.092		
Low C3, n (%)	60 (81.1)	214 (70.6)	1.991 (0.968-4.095)	0.061		
C4, mean ± SD, mg/dL	11.0 ± 6.8	13.9 ± 11.2	0.962 (0.929-0.997)	**0.035**		
Low C4, n (%)	37 (50.0)	117 (38.6)	1.610 (0.952-2.722)	0.076		
Serum creatinine, mean ± SD, μmol/L	82.4 ± 95.1	86.0 ± 59.0	0.999 (0.995-1.003)	0.681		
Urine protein, median (IQR), g/24 hour	2.0 (0.5-3.6)	2.1 (0.7-3.9)	0.949 (0.871-1.035)	0.241		
Prednisone dose, median (IQR), mg	20.0 (10.0-40.0)	30.0 (15.0-50.0)	0.995 (0.988-1.003)	0.208		
Hydroxychloroquine, n (%)	51 (68.9)	194 (64.0)	1.246 (0.722-2.149)	0.429		
Mycophenolate mofetil, n (%)	8 (10.8)	56 (18.5)	0.535 (0.243-1.177)	0.120		
Cyclophosphamide, n (%)	4 (5.4)	31 (10.2)	0.501 (0.171-1.467)	0.208		
Tacrolimus, n (%)	5 (6.8)	22 (7.3)	0.926 (0.338-2.531)	0.880		
Azathioprine, n (%)	5 (6.9)	12 (4.0)	1.757 (0.599-5.153)	0.304		
No immunosuppressants at present, n (%)	19 (25.7)	65 (21.5)	1.265 (0.702-2.280)	0.434		
ACE inhibitors or ARB, n (%)	44 (59.5)	220 (72.6)	0.553 (0.326-0.938)	**0.028**		

Ab, antibody; ACE, angiotensin converting enzyme; ARB, Aldosterone receptor blockers; C3, complement 3; C4, complement 4; DFS70, dense fine speckles 70; dsDNA, double-stranded DNA; IQR, interquartile range; LN, lupus nephritis; PCNA, proliferative cell nuclear antigen; Rib-p, ribosomal P protein; rSLEDAI, renal systemic lupus erythematosus disease activity index; SLEDAI-2K, systemic lupus erythematosus disease activity index-2000; SD, standard deviation; P value less than 0.05 is bold. †, Active LN means rSLEDAI ≥ 4.

**Table 2:** Correlations of O.D. values for anti-DFS70 antibodies with clinical parameters.

	*r* value	*P* value
Age	-0.076	0.142
Anti-dsDNA Ab	0.151	**0.003**
Anti-nucleosome Ab	0.170	**0.001**
C4	-0.116	**0.028**

Ab, antibody; C4, complement 4; DFS70, dense fine speckles 70; dsDNA, double-stranded DNA; O.D. value, optical density value; P value less than 0.05 is bold.

### Association of Anti-DFS70 Antibodies With Pathological Characteristics of LN Patients

Of the 377 LN patients, 82.0% (309/377) had PLN (38 class III; 121 class IV; 65 class III+V; 85 class IV+V), 15.6% (59/377) had MLN (class V), 2.1% (8/377) had early stage of LN (1 class I; 7 class II) and 0.3% (1/377) had end-stage renal disease (class VI). To identify the associated LN class for anti-DFS70 antibodies, we compared the distribution of LN class among patients with and without anti-DFS70 antibodies (**Table 3**). We observed 91.9% (68/74) of LN patients with positive anti-DFS70 antibodies had PLN, while in LN patients without anti-DFS70 antibodies, the frequency of PLN was 79.5% (241/303, *P*=0.013), which was significantly lower than LN patients with positive anti-DFS70 antibodies. Consistent with this, the positive rate of anti-DFS70 antibodies was higher in PLN (22.0%, 68/309) compared to MLN patients (10.2%, 6/59, *P*=0.0376). These data suggested the association of anti-DFS70 antibodies with PLN. In addition to anti-DFS70 antibodies, we also analyzed other clinically relevant parameters for PLN and compared them with MLN patients (**Supplemental Table 2**). We found that the covariates correlated with anti-DFS70 antibodies including anti-dsDNA, anti-nucleosome, and C4 (**Table 2**) which were also associated with PLN and not MLN patients (**Supplemental Table 2**). Given that the titer of anti-dsDNA antibodies had the highest accuracy in discriminating between PLN and MLN patients (16), we further analyzed the distribution of LN class in the 17 anti-dsDNA antibody-negative and anti-DFS70 antibody-positive LN patients, and we found that 88.2% (15/17) anti-dsDNA antibody-negative and anti-DFS70 antibody-positive LN was PLN patients, while 11.8% (2/17) was MLN patients.

**Table 3:** Distribution of anti-DFS70 antibodies in different LN class.

LN class	Positive anti-DFS70 antibodies n=74	Negative anti-DFS70 antibodies n=303	*P* value
Class I and II	0 (0.0)	8 (2.6)	0.36
Proliferative LN	68 (91.9)	241 (79.5)	**0.013**
Membrane LN	6 (8.1)	53 (17.5)	**0.046**
VI class	0 (0.0)	1 (0.3)	1.00

DFS70, dense fine speckles 70; LN, lupus nephritis; Proliferative LN included patients with class III, class IV, class III + V and class IV + V; Membranous LN referred to patients having pure class V LN. P value less than 0.05 is bold.

**Figure 2:**
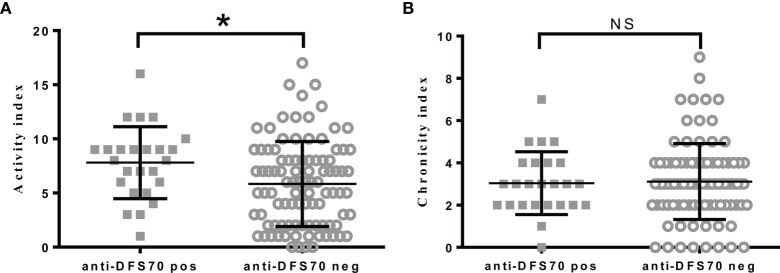
Comparisons of pathological activity and chronicity index in LN patients with positive or negative anti-DFS70 antibodies. **(A)** Comparison of activity index in LN patients with positive or negative anti-DFS70 antibodies; **(B)** Comparison of chronicity index in LN patients with positive or negative anti-DFS70 antibodies. DFS70, dense fine speckles 70; LN, lupus nephritis; pos, positive; neg, negative. *P < 0.05; NS, no significance.

Of the 121 LN patients that had AI and CI, the median (IQR) of the indexes were AI: 6.0 (3.0-9.0) and CI: 3.0 (2.0-4.0), respectively. To determine the association of anti-DFS70 antibodies with pathological activity status of LN patients, we divided LN patients into two groups based on whether they were positive or negative for anti-DFS70 antibodies, and then compared the differences of AI and CI between the groups. As shown in **Figure 2**, LN patients who were positive for anti-DFS70 antibodies had significantly higher AI [8.0 (5.5, 9.0) vs 6.0 (2.0, 8.0), *P*=0.0131, **Figure 2A**]. However, CI was similar between the groups [3.0 (2.0, 4.0) vs 3.0 (2.0, 4.0), *P*=0.8412, **Figure 2B**]. We then compared the clinical correlations of AI and CI in the 121 LN patients (**Supplemental Table 3**). We observed that the covariates correlated with anti-DFS70 antibodies including anti-dsDNA, anti-nucleosome, and C4 (**Table 2**) were also correlated with AI. Comparisons of the clinical correlations of AI in our study with previously published studies (24, 25) were summarized in **Supplemental Table 4**.

## Discussion

To our knowledge, the present study is the largest to date that determined the prevalence of anti-DFS70 antibodies in biopsy-proven LN patients. Our data demonstrated that LN patients had similar prevalence and O.D. values (**Figure 1**) for anti-DFS70 antibodies with those of NLN-SLE patients, and was consistent with several previous studies (5, 6, 10) where the prevalence of anti-DFS70 antibodies had no significant differences in SLE patients with or without nephritis. Although the treatment strategies (**Supplemental Table 1**) and disease duration of our LN and NLN-SLE patients were different, which was almost inevitable in clinical settings, a similar prevalence of anti-DFS70 antibodies in our newly onset LN and NLN-SLE patients was also be observed. Murine studies have demonstrated the protective effects of anti-DFS70 antibodies purified from HI in attenuating LN development in lupus-prone mice (11). However, our clinical observations do not support the protective role of anti-DFS70 antibodies in SLE patients.

In addition, our data demonstrated that the prevalence of anti-DFS70 antibodies in LN patients (19.6%) was significantly higher compared to CKD patients (13.4%) and age- and gender-matched HI (9.0%). This further suggests that anti-DFS70 antibodies are not protective in LN patients. There are a few possible reasons for the high prevalence of anti-DFS70 antibodies in these LN patients. First, detection assays and strategies could affect the detection of anti-DFS70 antibodies. This is because anti-DFS70 antibodies in LN patients are usually positive for anti-ENA and anti-dsDNA antibodies (5, 6, 8, 10). This could mask the DFS pattern of anti-DFS70 antibodies measured using indirect immunofluorescence assays (IFA), which use human epithelial type-2 cells (HEp-2) as substrates. Conversely, a positive anti-DFS70 antibody reading in HI is typically monospecific (8, 26). In our study, 30/31 anti-DFS70 antibody-positive CKD patients also had monospecific anti-DFS70 antibodies. A previous large cohort study (10) measured anti-DFS70 antibodies using a uniform chemiluminescence immunoassay and found that the prevalence of anti-DFS70 in their SLE patient cohort was higher compared to SLE patients from previous studies that used HEp-2 IFA to identify DFS pattern/anti-DFS70 antibodies (7.1% vs 0.0%-2.8%). In our study, anti-DFS70 antibodies were measured using uniform ELISA. We also observed that the prevalence of anti-DFS70 antibodies in LN (19.6%) was higher compared to previous studies (0.0%-2.8%) (10), while the prevalence of anti-DFS70 antibodies in HI (9.0%) was comparable to the frequency of DFS pattern in HI (7.8%), which was reported in our previous study (19).

Besides, ethnic differences may also contribute to the heterogeneous prevalence of anti-DFS70 antibodies in different countries (8–10). Choi et al. reported that SLE patients from Canada were less likely to have positive anti-DFS70 antibodies compared to patients of African descent (10). In our SLE patients, the prevalence of anti-DFS70 antibodies was higher compared to previous studies (5, 6, 10, 27, 28) that had patients of Caucasian descent from Turkey (0.3%-8.1%), the United States (2.97%-12.2%), and Canada (1.6%-4.9%), but was comparable with a Japanese study (22.1%) that comprised of patients with Asian descent (29).

We also observed that O.D. values for anti-DFS70 antibodies correlated with disease activity markers in LN patients (**Table 2**), which included anti-dsDNA antibodies, anti-nucleosome, and C4. The positive association of anti-DFS70 with anti-dsDNA antibodies remained significant after stepwise multi-variable analysis (**Table 1**). This suggested that anti-DFS70 antibodies had the potential to be a non-histological biomarker to reflect pathological activity in LN patients similar to anti-dsDNA antibodies. Choi et al. reported that SLE patients with positive anti-dsDNA antibodies were less likely to have anti-DFS70 antibodies (10), however, the lower disease activity and shorter disease duration of their SLE patients compared to LN patients in our study may be the reason for our differing results. In another one of our ongoing studies, we detected anti-DFS70 antibodies in long-term follow-up SLE patients which also suggested that the differences in anti-DFS70 antibodies titers were related to anti-dsDNA antibody titers.

Similar to anti-dsDNA, anti-DFS70 antibodies were more prevalent in PLN (22.0%) compared to MLN patients (10.2%). As a distinct form of LN, MLN histologically resembles PMN (30). We found that the prevalence of anti-DFS70 in MLN was also similar to PMN patients (11.4%). Compared to MLN, PLN patients in this study had higher titers of autoantibodies such as anti-dsDNA antibodies and worse renal function (**Supplemental Table 2**). This was consistent with previous observations (2). PLN is the most severe form of LN and is characterized by renal deposition of anti-dsDNA antibodies and a variety of antigens, together with the proliferation of mesangial and endothelial cells, which are active pathological manifestations observed in LN patients (2). Accordingly, our data showed that LN patients with positive anti-DFS70 had significantly higher AI (**Figure 2A**). Other pathological activity markers reported in previous studies (24, 25), such as anti-dsDNA, anti-nucleosome, and C3 (**Supplemental Table 4**) also correlated with AI instead of CI (**Supplemental Table 3**). Given that renal biopsies may not always be available in clinical settings, it is of clinical significance for anti-DFS70 antibodies to reflect pathological activity and LN subclass. The high frequency of PLN patients in anti-dsDNA antibody-negative and anti-DFS70 antibody-positive LN patients also suggested that anti-DFS70 antibodies could serve as an alternative PLN predictive biomarker in anti-dsDNA antibody-negative LN patients. Active PLN patients typically need powerful immunosuppressive treatment, while MLN patients are usually treated without intense immunosuppressive agents (2). Hence, detection of anti-DFS70 antibodies may contribute to the early therapeutic intervention in PLN patients. The association of anti-DFS70 antibodies with AI instead of clinical rSLEDAI may also be of pathological significance, considering the discrepancies observed in clinical and pathological activities in LN patients (31).

The limitation of this study was that longitudinal measurements of anti-DFS70 antibodies in our LN patients with high disease activity were not performed. Investigating the relationships of anti-DFS70 antibodies with remission of renal activity is also critical and should be considered in future studies.

In conclusion, our study found that the prevalence of anti-DFS70 antibodies in biopsy-proven LN patients was comparable to NLN-SLE patients. Furthermore, anti-DFS70 antibodies were associated with anti-dsDNA antibodies, renal AI, and PLN, which suggested that anti-DFS70 antibodies may not have a protective role in LN patients, but may have a potential role as a non-histological biomarker to reflect LN subclass and pathological activity.

## Data Availability Statement

The original contributions presented in the study are included in the article/**Supplementary Material**. Further inquiries can be directed to the corresponding authors.

## Ethics Statement

The studies involving human participants were reviewed and approved by the Institutional Review Board of Renji Hospital. Written informed consent for participation was not provided by the participants’ legal guardians/next of kin because: Serum samples were obtained from the residual samples in Clinical Laboratory Department, the requirement for informed consent was waived.

## Author Contributions

DC, YD, FD, EL, XN, and ZW collected serum samples and patient clinical data. LZ and YD performed the experiments. DC, BZ, and LL analyzed the data and wrote the manuscript. BZ and LL supervised the study and revised the manuscript. All authors contributed to this manuscript and approved the final version for publication.

## Funding

This study was funded by the National Key Research and Development Program of China (No. 2017YFC0909002), the National Natural Science Foundation of China (grants 81974251) and the Zhongnanshan Medical Foundation of Guangdong Province (No. ZNSXS-20220007).

## Conflict of Interest

The authors declare that the research was conducted in the absence of any commercial or financial relationships that could be construed as a potential conflict of interest.

## Publisher’s Note

All claims expressed in this article are solely those of the authors and do not necessarily represent those of their affiliated organizations, or those of the publisher, the editors and the reviewers. Any product that may be evaluated in this article, or claim that may be made by its manufacturer, is not guaranteed or endorsed by the publisher.
