# Supplementary material for: Anti-DFS70 Antibodies Are Associated With Proliferative Lupus Nephritis and Renal Pathological Activity
Source: Front Immunol. 2022 Feb 3;13:810639. doi: 10.3389/fimmu.2022.810639 (PMC8850646; doi:10.3389/fimmu.2022.810639)
Supplement: Supplementary file 1 [file DataSheet_1.docx]

**Supplemental table 1**. Comparisons of medications between NLN-SLE and LN patients

| Medication | NLN-SLE n=268 | LN  n=377 | *P* value |
| --- | --- | --- | --- |
| Prednisone dose, median (IQR), mg | 20 (10-40) | 30 (15-50) | **0.0117** |
| Hydroxychloroquine, n (%) | 147 (54.9) | 245 (65.0) | **0.0094** |
| Mycophenolate mofetil, n (%) | 21 (7.8) | 64 (17.0) | **0.0007** |
| Cyclophosphamide, n (%) | 3 (1.1) | 35 (9.3) | **0.0001** |
| Tacrolimus, n (%) | 6 (2.2) | 27 (7.2) | **0.0058** |
| Azathioprine, n (%) | 10 (3.7) | 17 (4.5) | 0.6935 |
| No immunosuppressants at present, n (%) | 93 (34.7) | 84 (22.3) | **0.0005** |
| ACE inhibitors or ARB, n (%) | 56 (20.9) | 264 (70.3) | **0.0001** |

ACE, angiotensin converting enzyme; ARB, Aldosterone receptor blockers; LN, lupus nephritis; NLN-SLE, non-LN systemic lupus erythematosus. *P* value less than 0.05 is bold.

**Supplemental table 2**. Comparisons of clinical characteristics between PLN and MLN patients

| Parameter | PLN  n=309 | MLN  n=59 | *P* value |
| --- | --- | --- | --- |
| Age, mean±SD, years | 37.0±12.5 | 37.7±12.9 | 0.700 |
| Gender, female, n (%) | 272 (88.0) | 54 (91.5) | 0.439 |
| Duration of SLE, median (IQR), years | 5.0 (1.4-10.0) | 3.0 (0.8-10.0) | 0.304 |
| rSLEDAI, median (IQR) | 8.0 (4.0-12.0) | 4.0 (4.0-8.0) | **0.0001** |
| Active LN†, n (%) | 257 (83.2) | 46 (78.0) | 0.323 |
| Anti-dsDNA Ab, mean±SD, IU/mL | 201.4±177.5 | 107.2±121.2 | **0.0001** |
| Anti-nucleosome Ab, mean±SD | 1.9±2.6 | 1.0±1.4 | **0.012** |
| Anti-C1q Ab, n (%) | 95 (30.7) | 10 (16.9) | **0.032** |
| Anti-C1q Ab, median (IQR), RU/mL | 9.9 (3.3-27.8) | 5.9 (2.3-17.4) | 0.071 |
| Anti-histone Ab, n (%) | 115 (37.2) | 10 (16.9) | **0.003** |
| Anti-Sm Ab, n (%) | 61 (19.7) | 13 (22.0) | 0.659 |
| Anti-nRNP/Sm Ab, n (%) | 114 (36.9) | 32 (54.2) | **0.010** |
| Anti-SSA/Ro60 Ab, n (%) | 172 (55.7) | 33 (56.6) | 0.902 |
| Anti-Ro52 Ab, n (%) | 153 (49.5) | 33 (55.9) | 0.324 |
| Anti-SSB/La Ab, n (%) | 26 (8.4) | 8 (13.6) | 0.201 |
| Anti-PCNA Ab, n (%) | 8 (2.6) | 2 (3.4) | 1.000 |
| Anti-Rib-p Ab, n (%) | 88 (28.5) | 21 (35.6) | 0.250 |
| C3, mean±SD, mg/dL | 65.6±28.7 | 87.5±34.7 | **0.0001** |
| Low C3, n (%) | 239 (77.3) | 30 (50.8) | **0.0001** |
| C4, mean±SD, mg/dL | 12.8±10.8 | 16.0±9.4 | **0.040** |
| Low C4, n (%) | 134 (43.4) | 18 (30.5) | **0.040** |
| Serum [creatinine](C:/Users/Laptop/AppData/Local/youdao/dict/Application/8.9.6.0/resultui/html/index.html#/javascript:;), mean±SD, μmol/L | 89.8±71.4 | 59.6±21.5 | **0.001** |
| Urine protein, median (IQR), g/24 hour | 2.2 (0.7-4.0) | 1.5 (0.6-3.1) | 0.295 |

Ab, antibody; C3, complement 3; C4, complement 4; DFS70, dense fine speckles 70; dsDNA, double-stranded DNA; IQR, interquartile range; PCNA, proliferative cell nuclear antigen; PLN, proliferative lupus nephritis; Rib-p, ribosomal P protein; rSLEDAI, renal systemic lupus erythematosus disease activity index; SD, standard deviation; MLN, membrane lupus nephritis. *P* value less than 0.05 is bold. †, Active LN means rSLEDAI ≥ 4.

**Supplemental table 3.** Clinical correlations of activity and chronicity index

|  | Activity index  *r* value (*P* value) | Chronicity index  *r* value (*P* value) |
| --- | --- | --- |
| Anti-dsDNA Ab, IU/mL | 0.326 (0.0001) | -0.057 (0.535) |
| Anti-nucleosome Ab titer | 0.304 (**0.001**) | -0.159 (0.085) |
| Anti-C1q Ab, RU/mL | 0.168 (0.066) | -0.186 (0.041) |
| Anti-DFS70 Ab | 0.225 (0.013) | -0.019 (0.840) |
| Anti-histone Ab | 0.256 (0.005) | -0.081 (0.383) |
| Anti-Sm Ab | -0.060(0.520) | -0.067 (0.468) |
| Anti-nRNP/Sm Ab | -0.172 (0.062) | -0.107 (0.249) |
| Anti-SSA/Ro60 Ab | 0.121 (0.193) | 0.046 (0.618) |
| Anti-Ro52 Ab | 0.013 (0.887) | 0.084 (0.365) |
| Anti-SSB/La Ab | -0.033 (0.725) | -0.013 (0.889) |
| Anti-PCNA Ab | -0.023 (0.803) | -0.104 (0.264) |
| Anti-Rib-p Ab | -0.145 (0.117) | -0.214 (0.020) |
| C3, mg/dL | -0.526 (0.0001) | 0.132 (0.158) |
| C4, mg/dL | -0.322 (0.0001) | 0.225 (0.016) |
| rSLEDAI | 0.418 (0.0001) | 0.051 (0.586) |

Ab, antibody; C3, complement 3; C4, complement 4; DFS70, dense fine speckles 70; dsDNA, double stranded DNA; PCNA, proliferative cell nuclear antigen; Rib-p, ribosomal P protein; rSLEDAI, renal systemic lupus erythematosus disease activity index; *P* value less than 0.05 is bold.

**Supplemental table 4.** Comparisons of clinical correlations of pathological activity index in our study versus two published studies.

|  | our study | Yang X-W et. al. (24) | Moroni G et. al. (25) |
| --- | --- | --- | --- |
| Anti-dsDNA Ab, IU/mL | ***↑ | *↑ | **↑ |
| Anti-nucleosome Ab titer | **↑ | / | *↑ |
| **Anti-C1q Ab, RU/mL** | NS | ***↑ | **↑ |
| Anti-DFS70 Ab | *↑ | / | / |
| Anti-histone Ab | **↑ | / | / |
| Anti-Sm Ab | NS | NS | / |
| **Anti-nRNP/Sm Ab** | NS | *↓ | / |
| Anti-SSA/Ro60 Ab | NS | NS | / |
| Anti-Ro52 Ab | NS | / | / |
| Anti-SSB/La Ab | NS | NS | / |
| Anti-PCNA Ab | NS | / | / |
| Anti-Rib-p Ab | NS | / | NS |
| C3, mg/dL | ***↓ | / | ***↓ |
| C4, mg/dL | ***↓ | / | **↓ |
| rSLEDAI | ***↑ | / | / |

Ab, antibody; C3, complement 3; C4, complement 4; DFS70, dense fine speckles 70; dsDNA, double-stranded DNA; PCNA, proliferative cell nuclear antigen; Rib-p, ribosomal P protein; rSLEDAI, renal systemic lupus erythematosus disease activity index; * *P* < 0.05, ** *P* < 0.01, *** *P* < 0.001; NS, no significance; /, No data; ↑, positively associated to activity index; ↓, negatively associated to activity index; Boldfaced characters were the events that showed differing tendencies in our study compared to the studies of Yang X-W et. al. (24) and Moroni G et. al. (25).
